# Cortical gyrification differences between early- and late-onset obsessive–compulsive disorder: neurobiological evidence for neurodevelopmentally distinct subtypes

**DOI:** 10.1017/S0033291722003129

**Published:** 2023-10

**Authors:** Inkyung Park, Minji Ha, Taekwan Kim, Silvia Kyungjin Lho, Sun-Young Moon, Minah Kim, Jun Soo Kwon

**Affiliations:** 1 Department of Brain and Cognitive Sciences, Seoul National University College of Natural Sciences, Seoul, Republic of Korea; 2 Department of Bio and Brain, Korea Advanced Institute of Science and Technology, Daejeon, Republic of Korea; 3 Department of Psychiatry, Seoul National University College of Medicine, Seoul, Republic of Korea; 4 Department of Neuropsychiatry, Seoul National University Hospital, Seoul, Republic of Korea; 5 Department of Psychiatry, Hallym University Kangnam Sacred Heart Hospital, Seoul, Republic of Korea; 6 Institute of Human Behavioral Medicine, SNU-MRC, Seoul, Republic of Korea

**Keywords:** Cortical folding, gyrification, neurodevelopment, obsessive–compulsive disorder, onset age, subtypes, verbal fluency, visuospatial memory

## Abstract

**Background:**

Identifying more homogenous subtypes of patients with obsessive–compulsive disorder (OCD) using biological evidence is critical for understanding complexities of the disorder in this heterogeneous population. Age of onset serves as a useful subtyping scheme for distinguishing OCD into two subgroups that aligns with neurodevelopmental perspectives. The underlying neurobiological markers for these distinct neurodevelopmental differences can be identified by investigating gyrification changes to establish biological evidence-based homogeneous subtypes.

**Methods:**

We compared whole-brain cortical gyrification in 84 patients with early-onset OCD, 84 patients with late-onset OCD, and 152 healthy controls (HCs) to identify potential markers for early neurodevelopmental deficits using the local gyrification index (lGI). Then, the relationships between lGI in clusters showing significant differences and performance in visuospatial memory and verbal fluency, which are considered trait-related neurocognitive impairments in OCD, were further examined in early-onset OCD patients.

**Results:**

The early-onset OCD patients exhibited significantly greater gyrification than those with late-onset OCD patients and HCs in frontoparietal and cingulate regions, including the bilateral precentral, postcentral, precuneus, paracentral, posterior cingulate, superior frontal, and caudal anterior cingulate gyri. Moreover, impaired neurocognitive functions in early-onset OCD patients were correlated with increased gyrification.

**Conclusions:**

Our findings provide a neurobiological marker to distinguish the OCD population into more neurodevelopmentally homogeneous subtypes, which may contribute to the understanding of the neurodevelopmental underpinnings of an etiology in early-onset OCD consistent with the accumulated phenotypic evidence of greater neurodevelopmental deficits in early-onset OCD than in late-onset OCD.

## Introduction

Despite advances in treatments, many obsessive–compulsive disorder (OCD) patients have reported poor treatment responses mainly due to heterogeneity of the disease (Lack, 2012; Rosa-Alcazar, Sanchez-Meca, Gomez-Conesa, & Marin-Martinez, 2008). OCD is considered clinically heterogeneous in that many OC symptoms, such as washing, checking, or ordering, may meet the diagnostic criteria for the disorder, and various other phenotypic differences are also evident in OCD patients (Taylor, 2011), making it difficult to identify pure characteristics of the disorder. The Research Domain Criteria project also addresses the need for the identification of more homogeneous subgroups of patients with psychiatric disorders using biological evidence (Insel, 2014; Wardenaar & de Jonge, 2013). To date, several useful subtyping methods have been proposed to identify more homogeneous OCD subgroups to better understand complexities of the disorder (Leckman et al., 2010), and evidence suggests that age of onset serves as an important subtyping scheme for reliably distinguishing OCD populations (Taylor, 2011).

The onset age of OCD is bimodally distributed, with an early peak in childhood or early adolescence before puberty and a late peak in early adulthood, suggesting distinct subtypes between early- and late-onset OCD (Geller et al., 1998; Swedo, Rapoport, Leonard, Lenane, & Cheslow, 1989). Early-onset OCD patients are known to have onset of subclinical symptoms before puberty, while late-onset OCD patients are likely to show obsessive and compulsive symptoms after puberty (Sobin, Blundell, & Karayiorgou, 2000). Using this criterion, 17 years has been suggested as a cutoff onset age for classifying two OCD patient subgroups approximately at puberty and has been used in many studies including our previous work (Butwicka & Gmitrowicz, 2010; Fontenelle, Mendlowicz, Marques, & Versiani, 2003; Henin et al., 2001; Hesse et al., 2011; Hwang et al., 2007; Kang, Kim, Kim, Hwang, & Kim, 2017; Kim et al., 2020; Wang et al., 2012). Previous systematic reviews and meta-analyses have reported that patients with early-onset OCD are reliably differentiated from those with late-onset OCD in terms of multiple etiologic and phenotypic factors, with early-onset OCD patients showing a higher prevalence in males, higher levels of genetic loading and heritability, higher comorbidity rates with other neurodevelopmental diseases such as tics and Tourette's syndrome, poorer treatment responses, and a more gradual appearance of symptoms than patients with late-onset OCD, suggesting that early-onset OCD patients have more neurodevelopmental loading than late-onset OCD patients (Geller et al., 1998; Taylor, 2011). In addition to these well-known clinical comparisons, neurobiological differences have also been evidenced in subtypes of OCD based on onset age (Boedhoe et al., 2017; Jurng et al., 2021; Kim et al., 2020), suggesting that there are distinct pathophysiological mechanisms in early- and late-onset OCD groups. However, the underlying neural markers to establish neurodevelopmental differences between patients with early- and late-onset OCD have rarely been investigated, although they need to be identified to subtype OCD patients into more biological evidence-based homogeneous subgroups to appropriately understand the neurobiological underpinnings of different etiologies.

Brain gyrification is mostly determined in the last two trimesters of pregnancy, and its pattern remains relatively stable throughout life, making it a reliable marker for early neurodevelopmental deficits in psychiatric illnesses (Armstrong, Schleicher, Omran, Curtis, & Zilles, 1995; Zilles, Palomero-Gallagher, & Amunts, 2013). Based on the concept that neurodevelopmental risk factors may play key roles in OCD pathophysiology (Huyser, Veltman, de Haan, & Boer, 2009; Rosenberg & Keshavan, 1998), gyrification has been previously investigated in OCD patients to determine whether early developmental alterations exist. However, previous findings on gyral patterns have been inconsistent, with some studies showing increased gyrification and others showing decreased gyrification, although the regions with abnormal gyrification were somewhat consistent in OCD patients. These conflicting findings were mainly due to differences in the methods used to measure gyrification. The first two studies reported reduced gyrification in the anterior cingulate gyrus (ACC) (Shim et al., 2009) and prefrontal cortex (Wobrock et al., 2010) in OCD patients compared to healthy controls (HCs) using a two-dimensional (2D) approach of assessing gyrification in regions of interest (ROIs). However, this previously adapted method has several limitations in that the surface perimeter is biased by slice orientation and inclusion of buried sulci, and it is impossible to precisely localize gyral abnormalities in sublobar regions, which raises questions of accuracy and reproducibility (Schaer et al., 2008).

Subsequent studies used three-dimensional (3D) vertexwise analyses of the local gyrification index (lGI), which enables a non-biased estimation of whole-brain vertexwise morphometric changes across all of the sulcogyral regions in the cerebral cortex. However, conflicting results continued to be reported. While Venkatasubramanian et al. (2012) found no significant differences using an ROI-based lGI approach, both Fan et al. (2013) and Rus et al. (2017) found hyper- and hypogyrification, respectively, in similar regions of the frontoparietal cortex using whole-brain lGI analysis. These heterogeneous results among lGI studies could be partly explained by several confounding factors, such as illness severity and medication status, because these factors could possibly affect cortical morphology (Fan et al., 2013). In these two previous studies using lGI in the whole-brain, which was the same method we used, only unmedicated or drug-naive OCD patients showed increased gyrification. Importantly, such inconsistent results raise the likelihood that there are neurodevelopmentally distinct subtypes in OCD, highlighting the significance of investigating gyrification differences in separate subgroups rather than investigating gyrification in a whole OCD population compared to HCs. Since abnormal gyrification reflects early neurodevelopmental cortical deficits (Armstrong et al., 1995; Zilles et al., 2013), investigating gyrification differences between the early- and late-onset OCD subgroups may help establish more biological evidence-based homogeneous subtypes in OCD that aligns with neurodevelopmental perspectives.

Furthermore, among the various neurocognitive function impairments reported in OCD (Shin, Lee, Kim, & Kwon, 2014), visual memory and verbal fluency deficits are persistently observed during pharmacological treatment (Kim, Park, Shin, & Kwon, 2002; Roh et al., 2005) and cognitive behavioral therapy (Vandborg, Hartmann, Bennedsen, Pedersen, & Thomsen, 2015) despite significant improvements in OC symptoms. That these neuropsychological deficits are still apparent in fully recovered OCD patients and their unaffected relatives (Rao, Reddy, Kumar, Kandavel, & Chandrashekar, 2008; Zartaloudi, Laws, & Bramon, 2019) further supports the hypothesis that these impairments are trait-related markers for OCD rather than state-dependent features of the disease. In fact, neuropsychological differences in visual memory and verbal fluency have been consistently reported between the early- and late-onset OCD subtypes (Hwang et al., 2007; Kim et al., 2020; Roth, Milovan, Baribeau, & O'Connor, 2005), suggesting that these deficits are prominent neurocognitive features reflecting effects of neurodevelopmental abnormalities on cortical gyrification that distinguish OCD into more homogenous subgroups.

In this study, we investigated cortical gyrification differences in OCD subgroups based on age of onset compared to HCs to identify potential neurobiological markers that can be used to subdivide neurodevelopmentally homogeneous subtypes in OCD. Since our OCD patients were all drug-naïve or unmedicated, and early-onset OCD patients are likely to have higher neurodevelopmental loading according to previous studies, it was hypothesized that patients with early-onset OCD would exhibit hypergyrification in frontoparietal and cingulate regions compared to late-onset OCD patients and HCs. Furthermore, to strengthen the evidence regarding the effects of neurodevelopmental deficits on abnormal gyrification, neurocognitive functions, such as visuospatial memory and verbal fluency, which are suggested to be trait-related impairments in OCD and prominent neurocognitive features that show differences between neurodevelopmental subtypes of OCD, were compared among OCD subgroups and the HC group, and the associations between these neurocognitive impairments and clusters with significant differences in gyrification were explored in exploratory analyses.

## Methods

### Participants

A total of 177 OCD patients and 152 HCs participated in this study, and their T1 magnetic resonance imaging (MRI) data were used in our previous studies (Jurng et al., 2021; Kim et al., 2020). Patients with OCD were recruited from the OCD clinic of the Department of Psychiatry at Seoul National University Hospital (SNUH, Seoul, South Korea). Patients were diagnosed with OCD based on the *Diagnostic and Statistical Manual of Mental Disorders*, fourth edition (DSM-IV) by experienced psychiatrists. The severity of obsessive–compulsive symptoms was evaluated in patients using the Yale–Brown Obsessive Compulsive Scale (Y-BOCS) (Goodman et al., 1989). OCD patients were also administered with the Hamilton Depression Rating Scale (HAM-D) (Hamilton, 1960) and the Hamilton Anxiety Rating Scale (HAM-A) (Hamilton, 1959) to assess the severity of depression and anxiety, respectively. All patients were either drug-naïve or unmedicated for at least 1 month at the time of inclusion (78 drug-naïve and 99 unmedicated). We designated the cutoff onset age to be 17 years, classifying the two OCD subgroups approximately at puberty to be consistent with previous studies: early-onset OCD (age at onset <17 years; *n* = 84) and late-onset OCD (age at onset >17 years; *n* = 84) (de Mathis et al., 2008). Data from nine OCD patients with an onset age of 17 years were excluded from further analysis. Psychiatric comorbidities were assessed according to the International Classification of Diseases, 10th edition (ICD-10): 6 OCD patients had a diagnosed comorbidity of major depressive disorder; 2 patients had dysthymic disorder; 39 patients had depressive disorder, not otherwise specified (NOS); 3 patients had bipolar II disorder; 1 patient had bipolar disorder, NOS; 4 patients had tic disorder; and 10 patients had personality disorders. HCs were recruited via internet advertisements and screened for the presence of psychiatric disorders or symptoms using the Structured Clinical Interview for DSM-IV–Non-Patient Version (SCID-NP). Potential HCs were excluded if they reported past or current axis-I diagnoses. The intelligence quotient (IQ) of all participants was evaluated with the Korean version of the Wechsler Adult Intelligence Scale (K-WAIS) to estimate intelligence. The exclusion criteria for both the OCD and HC groups included a lifetime history of any major psychotic disorders, substance abuse or dependence (except nicotine), neurological disease or clinically important head injury, and intellectual disability (IQ < 70).

Written informed consent was obtained from all participants and from the parents of subjects under 18 years of age after the procedures had been fully explained (IRB nos. H-1201-008-392, H-1110-009-380, and H-1503-045-655). The present study was also approved by the Institutional Review Board at Seoul National University Hospital (IRB no. H-2101-053-1188).

### Neurocognitive function tests

Visuospatial memory and verbal fluency, among the neurocognitive functions that were previously reported to reveal abnormalities in OCD patients compared to HCs (Shin et al., 2014), were examined in 71 early-onset OCD patients, 71 late-onset OCD patients, and 98 HCs.

Visuospatial memory was evaluated using the following Rey–Osterrieth Complex Figure Test (RCFT) (Meyers & Meyers, 1995) subsets: immediate recall (IR; 3 min after the copy condition) and delayed recall (DR; 30 min after the copy condition) conditions. The method developed by Meyers and Meyers was used to rate response times and accuracy in each condition (Meyers & Meyers, 1995).

To assess verbal fluency, the Korean version of the Controlled Oral Word Association Test (COWA) (Kang, Chin, Na, Lee, & Park, 2000) was administered. Within 1 min, subjects were required to remember as many words as possible starting with a given letter and belonging to a specific category. The total number of responses for each letter and category was counted and scored.

### Image acquisition

High-resolution anatomical T1-weighted (T1) scans were acquired using a Siemens 3T MAGNETOM Trio MRI scanner (Siemens, Erlangen, Germany) from all subjects. A 12-channel head coil and a 3D magnetization-prepared rapid acquisition gradient-echo (MPRAGE) sequence were used for the T1 images with the following imaging acquisition parameters: repetition time (TR), 1670 ms; echo time (TE), 1.89 ms; flip angle, 9°; field of view, 250 mm; voxel size, 1.0 × 0.98 × 0.98 mm^3^; and 208 slices. For quality control, the obtained MRI images were visually inspected for any artifacts or malformation of brain structures.

### Image processing

The preprocessing of the structural T1 images was performed using the FreeSurfer software package (version 5.3.0, http://surfer.nmr.mgh.harvard.edu) in accordance with the standard and automatic reconstruction algorithm (Fischl & Dale, 2000). This processing stream consists of removal of non-brain tissue, automated transformation to Talairach space, intensity normalization, and segmentation of gray/white matter tissue resulting in a white mesh and a pial mesh. The meshes consist of approximately 150 000 vertices for each hemisphere.

Next, to measure gyrification, the lGI for the entire cortex was computed at each vertex with the FreeSurfer pipeline using the method of Schaer et al. (2008). This method is a vertexwise extension of the classic 2D GI approach, which is the ratio of the inner pial surface to the outer perimeter on coronal sections (Zilles, Armstrong, Schleicher, & Kretschmann, 1988). The 3D lGI method takes into account the inherent nature of the cortex, which includes buried sulci, and is not restricted by sulcal walls or biased by the orientation or thickness of the slices. This method calculates the amount of cortex buried within the sulcal folds compared with the amount of visible cortex across the whole brain, allowing for the region-specific localization of gyral deficits (Schaer et al., 2008).

In brief, the automatic lGI computation creates an outer smoothed surface tightly wrapping the pial surface and estimates 800 overlapping 25 mm spherical 3D ROIs on the smoothed outer surface and their corresponding paired circular ROIs on the pial surface. Then, the lGI at each vertex of the reconstructed cortical surface was measured as the ratio between the smoothed outer perimeter and inner buried contour of the cortex resulting in individual gyrification maps.

### Statistical analysis

Demographic variables between the early-onset OCD, late-onset OCD, and HC groups were compared using χ^2^ tests or one-way analysis of variance, and the neurocognitive variables were compared using analysis of covariance (ANCOVA) with age as a covariate, followed by post hoc Bonferroni tests, in Statistical Package for Social Sciences (IBM SPSS Version 22). Clinical variables were compared between the early- and late-onset OCD groups with χ^2^ tests or independent *t* tests.

A whole-brain lGI analysis for T1 images was conducted with FreeSurfer. The vertexwise lGI values were mapped on the average template subject (fsaverage) for each participant to generate the contrasts of the analysis in the Query Design Estimate Contrast (QDEC) tool in FreeSurfer. A general linear model for surface-based group analyses controlling for the effect of age as a covariate was conducted to analyze regional differences in gyrification between groups (discrete factors) at each vertex in both hemispheres. The lGI maps were then smoothed with a full-width at half-maximum Gaussian kernel of 5 mm. The cluster-forming threshold of significance was set to *p* < 0.05, and statistical corrections of clusters for multiple comparisons were conducted by Monte Carlo simulation with 10 000 permutations implemented in FreeSurfer. Sensitivity analyses were performed to control for the effect of duration of illness (DOI) by adding it as an additional covariate. Detailed information on these analyses is included in the Supplementary Methods in the online Supplementary materials.

Pearson's correlation analyses between mean lGI values of clusters showing significant group differences and neurocognitive function test scores were performed in early-onset OCD patients in an exploratory manner.

## Results

### Participant characteristics

The demographic and clinical characteristics and neurocognitive function test results of the participants are presented in Table 1. There were no significant group differences between groups except for age (*F* = 26.945, *p* < 0.001). Based on post hoc results, the early-onset OCD group was younger than both the late-onset OCD group (*p* < 0.001) and HCs (*p* = 0.021), and the late-onset OCD group was older than the HCs (*p* < 0.001). Early-onset OCD patients had a significantly lower onset age (*t* = −16.188, *p* < 0.001) (online Supplementary Fig. S1) and longer DOI (*t* = 5.076, *p* < 0.001) than late-onset OCD patients. No significant differences in clinical variables, such as symptom severity for obsession and compulsion, depression, and anxiety, and in comorbidities were found between the early- and late-onset OCD groups. Regarding the neurocognitive function test results, there were significant group differences in the RCFT IR (*F* = 4.615, *p* = 0.011) and DR (*F* = 3.631, *p* = 0.028) measures and the COWA letter (*F* = 5.473, *p* = 0.005) and category (*F* = 6.845, *p* = 0.001) measures based on ANCOVA with age as a covariate. Based on Bonferroni post hoc analysis results, the two OCD subgroups performed significantly worse than the HC group in the following neurocognitive tests: RCFT IR (*p* = 0.014), RCFT DR (*p* = 0.032), COWA letter (*p* = 0.010), and COWA category (*p* = 0.005) in the early-onset OCD group; COWA letter (*p* = 0.043) and COWA category (*p* = 0.012) in the late-onset OCD group.

**Table 1.** Characteristics of the participants

Demographic and clinical variables	Early-onset OCD (*n* = 84)	Late-onset OCD (*n* = 84)	HCs (*n* = 152)	*F/t/*χ^2^	*p*	Post hoc test
Age (years)	21.85 ± 5.25	28.58 ± 6.87	24.09 ± 6.09	26.945	<0.001	EO < HCs***; HCs < LO***; EO < LO***
Sex (male/female)	60/24	53/31	100/52	1.388	0.500	–
Handedness (right/left)	71/6	72/6	140/12	0.003	0.998	–
IQ	109.58 ± 9.98	112.61 ± 12.55	112.77 ± 11.88	2.086	0.126	–
Age of onset (years)	13.10 ± 2.19	23.80 ± 5.65	–	−16.188	<0.001	EO < LO***
DOI (years)	8.75 ± 5.73	4.78 ± 4.29	–	5.076	<0.001	LO < EO***
Drug-naïve/unmedicated	32/52	42/42	–	2.415	0.120	–
Y-BOCS score			–			–
Total	25.68 ± 6.44	24.43 ± 6.46	–	1.255	0.211	–
Obsessions	13.71 ± 3.48	12.99 ± 3.25	–	1.397	0.164	–
Compulsions	11.96 ± 3.97	11.44 ± 4.52	–	0.798	0.426	–
HAM-A score	9.76 ± 6.78	10.03 ± 5.16	–	−0.285	0.776	–
HAM-D score	10.30 ± 6.95	11.19 ± 5.89	–	−0.872	0.384	–
Comorbidity			–			–
Major depressive disorder	3/81	3/81	–	0.000	1.000	–
Dysthymic disorder	1/83	1/83	–	0.000	1.000	–
Depressive disorder, NOS	18/66	21/63	–	0.301	0.584	–
Bipolar II disorder	3/81	0/84	–	3.055	0.081	–
Bipolar disorder, NOS	1/83	0/84	–	1.006	0.316	–
Tic disorder	2/82	2/82	–	0.000	1.000	–
Personality disorders	4/80	6/78	–	0.425	0.514	–
ICV (mm^3^)	1 637 385.39 ± 129 845.70	1 595 024.42 ± 139 865.54	1 598 456.71 ± 157 732.39	2.350	0.097	–
*Neurocognitive performance*						
Visual memory						
RCFT IR	12.35 ± 3.44	13.45 ± 2.65	13.70 ± 2.86	4.615	0.011	EO < HCs*
RCFT DR	12.28 ± 3.18	13.21 ± 2.63	13.46 ± 2.80	3.631	0.028	EO < HCs*
Verbal fluency						
COWA letter	39.85 ± 10.89	39.79 ± 12.30	44.84 ± 10.20	5.473	0.005	EO < HCs*; LO < HCs*
COWA category	37.68 ± 9.15	39.00 ± 8.47	42.31 ± 8.95	6.845	0.001	EO < HCs**; LO < HCs*

OCD, obsessive–compulsive disorder; IQ, intelligence quotient; Y-BOCS, Yale–Brown Obsessive Compulsive Scale; HAM-A, Hamilton Anxiety Rating Scale; HAM-D, Hamilton Depression Rating Scale; NOS, not otherwise specified; ICV, intracranial volume; RCFT, Rey–Osterrieth Complex Figure Test; COWA, Controlled Oral Word Association Test; EO, early-onset OCD; LO, late-onset OCD; HCs, healthy controls.

Data are presented as the mean ± standard deviation.

*p < 0.05; **p < 0.01; ***p < 0.001.

### Group comparisons in gyrification

**Fig. 1.**
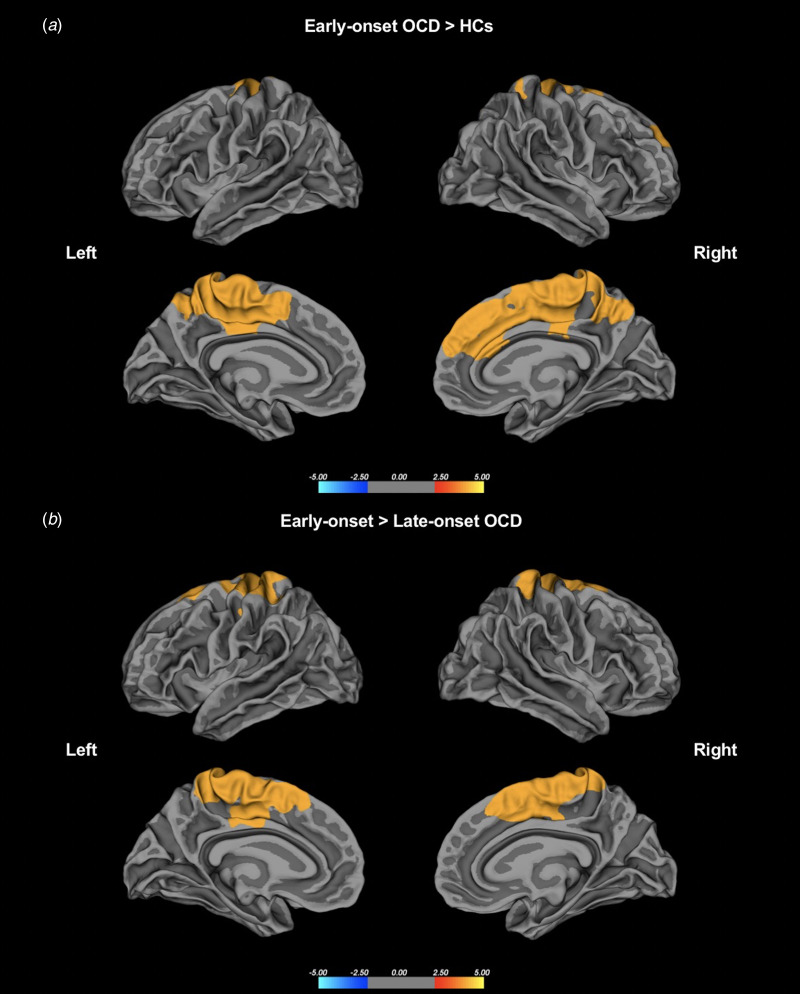
(a) Group differences in the whole-brain lGI between the patients with early-onset OCD and HCs. (b) Group differences in lGI between the patients with early-onset OCD and those with late-onset OCD. Statistical maps of the left and right hemispheres are shown in the lateral and medial views, respectively. The maps are shown for the clusters with significantly increased lGI in the patients with early-onset OCD after clusterwise correction for multiple comparisons (*p* < 0.05).

The results of the whole-brain lGI analysis are summarized in Tables 2 and 3. Significantly increased gyrification was observed in patients with early-onset OCD compared to HCs across several regions of the cortex with age as a covariate, including one cluster in the left hemisphere and one cluster in the right hemisphere. The left cluster with a significantly increased lGI (*p* < 0.001) had a peak vertex located within the precuneus gyrus and covered portions of the precentral, postcentral, precuneus, paracentral, posterior cingulate, superior frontal, and caudal anterior cingulate gyri. The right cluster with a significantly increased lGI (*p* < 0.001) had a peak vertex located within the paracentral gyrus and comprised of portions of the precentral, postcentral, precuneus, paracentral, posterior cingulate, superior frontal, and caudal anterior cingulate gyri. No region had a significantly reduced lGI in the early-onset OCD group compared to the HC group (Fig. 1a).

**Table 2.** Description of clusters with a significantly increased lGI in patients with early-onset OCD compared to HCs in the left and right hemispheres after clusterwise correction for multiple comparisons using Monte Carlo simulation (*p* < 0.05)

Cluster number	Peak vertex cluster	VtxMax	Size (mm^2^)	Peak vertex MNI			CWP (*p*)
				*x*	*y*	*z*	
1	L precuneus	124 011	4688.34	−15.8	−40.3	57.1	<0.001***
2	R paracentral	66 827	7317.53	6.6	−9.7	48.4	<0.001***

L, left hemisphere; R, right hemisphere; VtxMax, number of peak vertices in the significant cluster; MNI, Montreal Neurological Institute (coordinate system); CWP, clusterwise probability and the nominal *p* value.

***p < 0.001.

**Table 3.** Description of clusters with a significantly increased lGI in patients with early-onset OCD compared to patients with late-onset OCD in the left and right hemispheres after clusterwise correction for multiple comparisons using Monte Carlo simulation (*p* < 0.05)

Cluster number	Peak vertex cluster	VtxMax	Size (mm^2^)	Peak vertex MNI			CWP (*p*)
				*x*	*y*	*z*	
1	L precuneus	124 009	6711.65	−16.0	−38.8	57.4	<0.001***
2	R precentral	54 389	4999.35	11.8	−15.6	66.9	<0.001***

L, left hemisphere; R, right hemisphere; VtxMax, number of peak vertices in the significant cluster; MNI, Montreal Neurological Institute (coordinate system); CWP, clusterwise probability and the nominal *p* value.

***p < 0.001.

Significantly increased gyrification was also observed in patients with early-onset OCD compared to patients with late-onset OCD in similar regions, and these clusters had a peak vertex within the left precuneus and right precentral gyri and covered the precentral, postcentral, precuneus, paracentral, posterior cingulate, superior frontal, and caudal anterior cingulate gyri in both hemispheres. No region had a significantly reduced lGI in early-onset OCD patients compared to late-onset OCD patients (Fig. 1b). There were no significant differences between patients with late-onset OCD and HCs. Sensitivity analyses controlling for age and DOI as potential confounding effects showed similar regions of significance in early-onset OCD as the effects found in the main analyses, indicating that DOI did not confound the main results. Details on the regions that remained significant are included in the Supplementary Results, Fig. S2, and Table S1 in the online Supplementary materials.

### Associations between lGI and neurocognitive variables

**Fig. 2.**
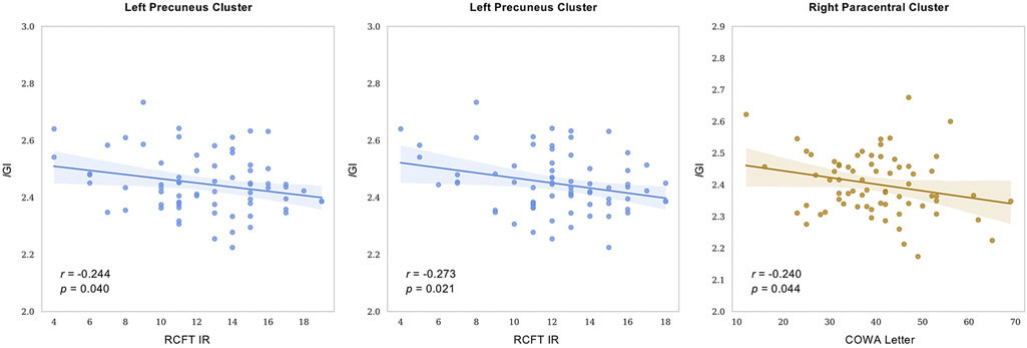
Correlations between the mean lGI values in clusters with significant differences between patients with early-onset OCD and HCs and neurocognitive function test results in the early-onset OCD patients. RCFT, Rey–Osterrieth Complex Figure Test; IR, immediate recall; DR, delayed recall; COWA, Controlled Oral Word Association Test.

The significantly increased mean lGI values showed correlations with poor neurocognitive function test scores in the early-onset OCD group (Fig. 2). Although these correlations did not survive after multiple comparisons, uncorrected *p* values are presented as an exploratory analysis. Patients with early-onset OCD showed significant negative correlations between the mean lGI in the left precuneus cluster and both RCFT IR (*r* = −0.244, *p* = 0.040) and RCFT DR (*r* = −0.273, *p* = 0.021) measures. A negative correlation between the significantly increased mean lGI in the right paracentral cluster and COWA letter scores (*r* = −0.240, *p* = 0.044) was also observed in the early-onset OCD group.

## Discussion

To identify underlying neurobiological markers that reflect the neurodevelopmental distinctions between OCD subgroups based on age of onset, we investigated gyrification differences between patients with early- and late-onset OCD who were drug-naïve or unmedicated. In this first study reporting gyrification differences in subtypes of OCD using whole-brain lGI, patients with early-onset OCD showed significantly increased gyrification patterns in medial and lateral frontoparietal and cingulate regions bilaterally when compared to those with late-onset OCD and HCs, while the late-onset OCD and HC groups showed no gyrification differences, which was consistent with the notion of greater neurodevelopmental deficits in early-onset OCD patients than those with late-onset OCD. Furthermore, impaired visuospatial memory and verbal fluency were related to higher lGI in both the left precuneus and right paracentral clusters in patients with early-onset OCD, supporting the idea that hypergyrification in frontoparietal and cingulate regions as a potential neurobiological marker may expand upon previous evidence of the neurodevelopmental model of early-onset OCD.

Cortical gyrification is regarded as a marker for early neurodevelopmental defects because its pattern is mainly formed and determined in the last two trimesters of pregnancy and remains relatively stable after birth (Armstrong et al., 1995; Zilles et al., 1988, 2013). In fact, gyrification is known to be more resistant to longitudinal changes and state-dependent effects of illness or medication than other structural parameters (Zilles et al., 2013). In the present study, the early-onset OCD group showed increased gyrification in similar regions when compared to the HC and late-onset OCD groups, while no differences were observed when the entire OCD group was compared to the HC group. Our results are in line with previous findings that reported hypergyrification in the middle frontal cortex extending to the precuneus region in drug-naïve or unmedicated OCD patients (Fan et al., 2013), and it can be inferred that the areas with significant neurodevelopmental differences in this previous study became more evident when OCD patients were subgrouped into neurodevelopmental subtypes and compared to HCs in our study. Our results of hypergyrification in early-onset OCD patients compared to that in late-onset OCD patients in the absence of symptom differences between the two subgroups further support the existence of distinct neurodevelopmental risk factors in the pathogenesis of early-onset OCD that may be predisposing to symptoms of the disorder and may serve as a potential structural marker for the neural correlates of OCD subtypes reflecting neurodevelopmental changes.

In this study, early-onset OCD patients had significantly increased gyrification in the frontoparietal and cingulate cortex, which are known as major component regions in the cortico–striato–thalamo–cortical (CSTC) model of OCD pathophysiology (Milad & Rauch, 2012). Although not directly comparable due to methodological differences, the regions of hypergyrification observed in our study are consistent with other neuroimaging findings, as earlier onset age in OCD was reported to be associated with larger precentral and middle frontal volumes (Kim et al., 2020) as well as medial frontal volumes (Koprivova et al., 2009). In addition, abnormal volume in the ACC was observed in an early illness state, and its lack of association with illness duration further suggested a neurodevelopmental basis for ACC abnormalities in OCD (Rosenberg & Keshavan, 1998). The alterations in gyrification are believed to reflect disruptions in underlying white matter connectivity of closely related cortical regions according to a tension-based model (Toro & Burnod, 2005; Van Essen, 1997; White, Su, Schmidt, Kao, & Sapiro, 2010). In the CSTC neuronal circuit, frontoparietal and cingulate regions are connected to the striatum and thalamus (Jung et al., 2014), and ventral prefrontal–striatal deficits in the CSTC have been proposed as neurodevelopmentally mediated network abnormalities in pediatric OCD patients (Huyser et al., 2009; Rosenberg & Keshavan, 1998). Reduced white matter integrity in arcuate fibers near the superior parietal lobule and decreased fiber quantities and thickness in highly connected cortical areas, including the postcentral, precuneus, posterior cingulate, paracentral, and several frontal regions, were reported as potential endophenotypes for OCD (Peng et al., 2014). Additionally, reduced myelin-related growth in both gray and white matter of the dorsolateral and medial frontal cortex centered around the superior frontal gyrus and ACC was further reported to be associated with psychiatric traits of compulsivity during cortical development in healthy subjects, suggesting alterations in cortical gyrification and structurally connected white matter in the frontoparietal and cingulate regions represent trait characteristics of OCD (Ziegler et al., 2019).

In the correlation analyses performed with an exploratory purpose, visuospatial memory and verbal fluency deficits were significantly associated with a higher mean lGI in early-onset OCD patients, supporting the notion that increased gyrification is a reliable marker for distinguishing neurodevelopmentally homogenous subtypes of OCD. Among the impairments in various neurocognitive functions observed in OCD, visual memory has been reported to show the largest effect size, with the most consistent reports of deficits in immediate recall in the RCFT (Shin et al., 2014). OCD patients have also shown persistent impairments in the RCFT and COWA during pharmacological treatments (Kim et al., 2002; Roh et al., 2005) and cognitive behavioral therapy (Vandborg et al., 2015) despite significant improvements in their OC symptoms. These neuropsychological deficits evidenced in fully recovered OCD patients and their unaffected relatives (Rao et al., 2008; Zartaloudi et al., 2019) further supported the hypothesis that impairments in visual memory and verbal fluency are trait markers for OCD rather than state-dependent features of the disease. In fact, neuropsychological differences in visual memory and verbal fluency have been consistently reported between early- and late-onset OCD subtypes. Hwang et al. (2007) observed worse visual memory and verbal fluency in late-onset OCD patients than in HCs, while Roth et al. (2005) reported worse visual memory and verbal fluency in late- and early-onset OCD patients, respectively, than in HCs. These inconsistent findings may have resulted from low general intelligence, medication effects, or different criteria for onset age (Hwang et al., 2007; Kim et al., 2020). However, in our previous volumetric study, with 17 years as the cutoff age of onset, unmedicated or drug-naïve early-onset OCD patients exhibited worse visuospatial memory than HCs (Kim et al., 2020). Using a larger sample, early-onset OCD patients in the current study demonstrated significant impairments in both RCFT and COWA performance in relationship with gyrification abnormalities, suggesting that visuospatial memory and verbal fluency deficits are prominent neurocognitive features for the neurodevelopmental subtyping of OCD.

### Limitations

There are several limitations that should be considered in this study. First, there is presently no clear standard for the cutoff onset age to distinguish between early- and late-onset OCD, and it is possible that the results would differ if the specific cutoff used by researchers to create such classification varies; thus, assumptions regarding onset age should be considered with caution before accepting the generality of the results. Second, the use of cross-sectional samples precludes investigation of the causal relationships between gyrification abnormalities as a vulnerability marker and the development of OCD. Thus, longitudinal MRI analyses in both early- and late-onset OCD patients are required to further clarify the neurodevelopmental origin and identify the longitudinal trajectories of these alterations in cortical folding. Third, the results of correlations between lGI and neurocognitive impairments were not corrected by multiple comparison and should be considered exploratory. In future studies, larger sample sizes would help to improve effect size and reproducibility of brain structure and cognitive phenotype correlations, and more pronounced effects of neurodevelopmental abnormalities may be seen with larger sample sizes (Marek et al., 2022).

## Conclusion

Our findings demonstrated that the early-onset OCD group had increased gyrification in the frontoparietal and cingulate gyri, key regions involved in the major pathophysiology of OCD, compared to the late-onset OCD and HC groups that was associated with impaired trait-related neurocognitive functions, which is consistent with the notion of greater neurodevelopmental deficits in early-onset OCD than in late-onset OCD. Taken together with previous findings of accumulated phenotypic and clinical evidence suggesting that early-onset OCD is a neurodevelopmental subtype of OCD, our findings provide biological evidence to distinguish the OCD population into more neurodevelopmentally homogeneous subtypes that may provide structural markers for understanding neurobiological underpinnings of different etiologies and helping to find appropriate treatment options for OCD subtypes.

## Supplementary material

For supplementary material accompanying this paper visit https://doi.org/10.1017/S0033291722003129. click here to view supplementary material
