# Supplementary material for: Cortical gyrification differences between early- and late-onset obsessive–compulsive disorder: neurobiological evidence for neurodevelopmentally distinct subtypes
Source: Psychol Med. 2022 Oct 19;53(13):5976–85. doi: 10.1017/S0033291722003129 (PMC10520599; doi:10.1017/S0033291722003129)
Supplement: Supplementary file 1 [file S0033291722003129sup001.docx]

Supplementary materials

**Cortical gyrification differences between early- and late-onset obsessive–compulsive disorder: Neurobiological evidence for neurodevelopmentally distinct subtypes**

Inkyung Park, Minji Ha, Taekwan Kim, Silvia Kyungjin Lho, Sun-Young Moon, Minah Kim, and Jun Soo Kwon

**Supplementary Methods**

Sensitivity analyses were performed to additionally control for duration of illness (DOI) to test whether the main findings were regionally specific. We repeated the main analysis of the whole-brain local gyrification index (lGI), while DOI was added as an additional covariate to test for potential confounding effects in Query Design Estimate Contrast (QDEC), while smoothing with a full-width at half-maximum (FWHM) of 5 mm after clusterwise correction for multiple comparisons (*p* < 0.05).

**Supplementary Results**

In sensitivity analyses controlling for age and DOI as potential confounding effects, the early-onset obsessive–compulsive disorder (OCD) patients showed similar regions of significance as the effects found in the main analyses. The early-onset OCD patients showed significantly higher gyrification than the late-onset OCD patients; the significant region was the same but relatively smaller compared to the regions of significance in the main results controlling only for age. This result was mainly due to the redundant controlling effect of age, which is already imbedded in the concept of DOI (= current age – onset age). These sensitivity analysis results controlling for age and DOI as potential confounding effects replicated in the early-onset OCD patients indicated that DOI did not confound the main results. Detailed results for the specific regions that remained significant are shown in Figure S2 and Table S1.

**Supplementary Figures**

**Figure S1.** Distribution of the onset age among those in the early- and late-onset OCD groups.


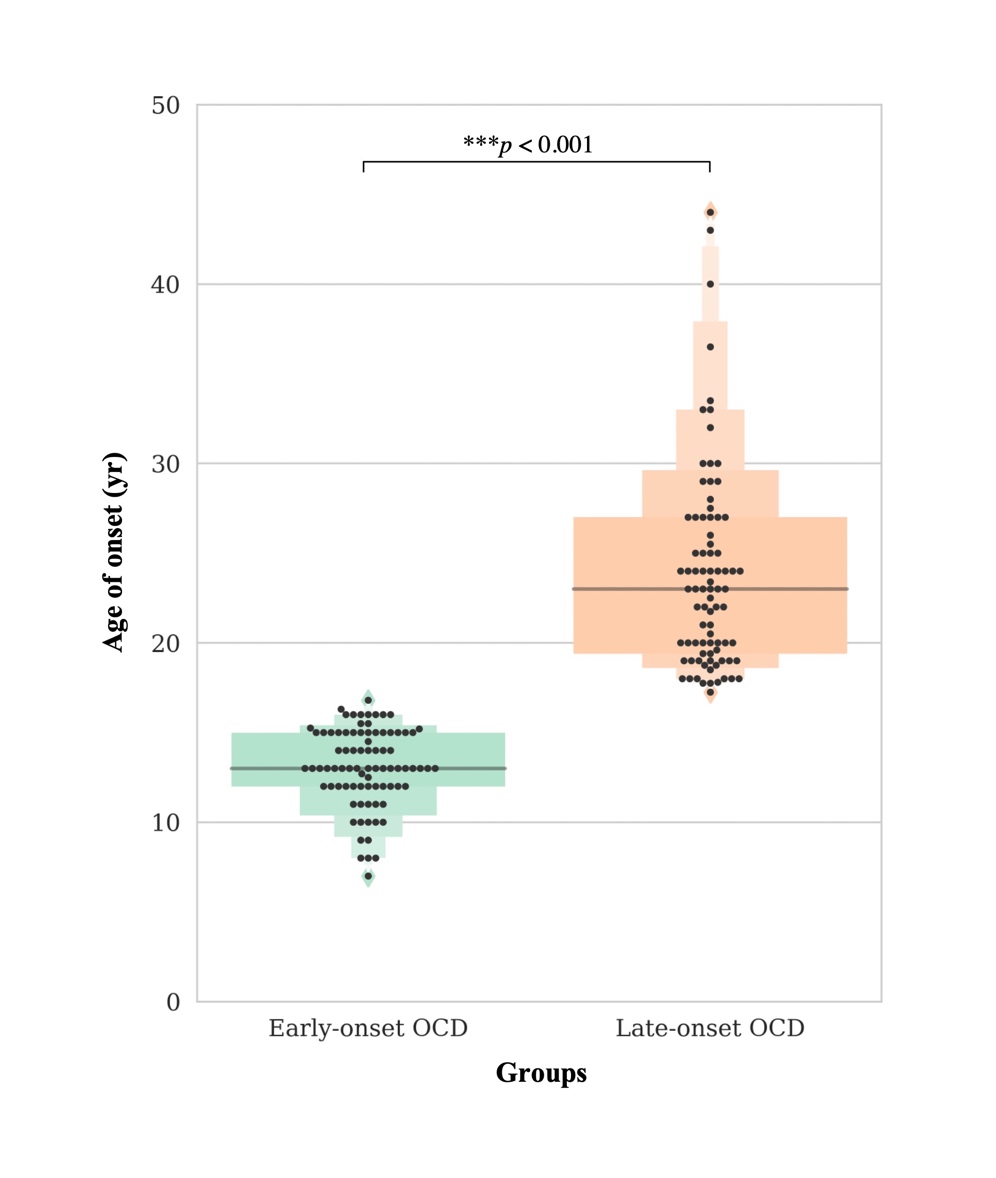


**Figure S2.** Group differences in the local gyrification index (lGI) between the patients with early-onset obsessive–compulsive disorder (OCD) and those with late-onset OCD. Statistical maps of the left and right hemispheres are shown in the lateral and medial views, respectively. The maps are shown for the clusters with significantly increased lGI in the patients with early-onset OCD controlling for both age and duration of illness (DOI) as covariates and smoothing with a full-width at half-maximum (FWHM) of 5 mm after clusterwise correction for multiple comparisons (*p* < 0.05).


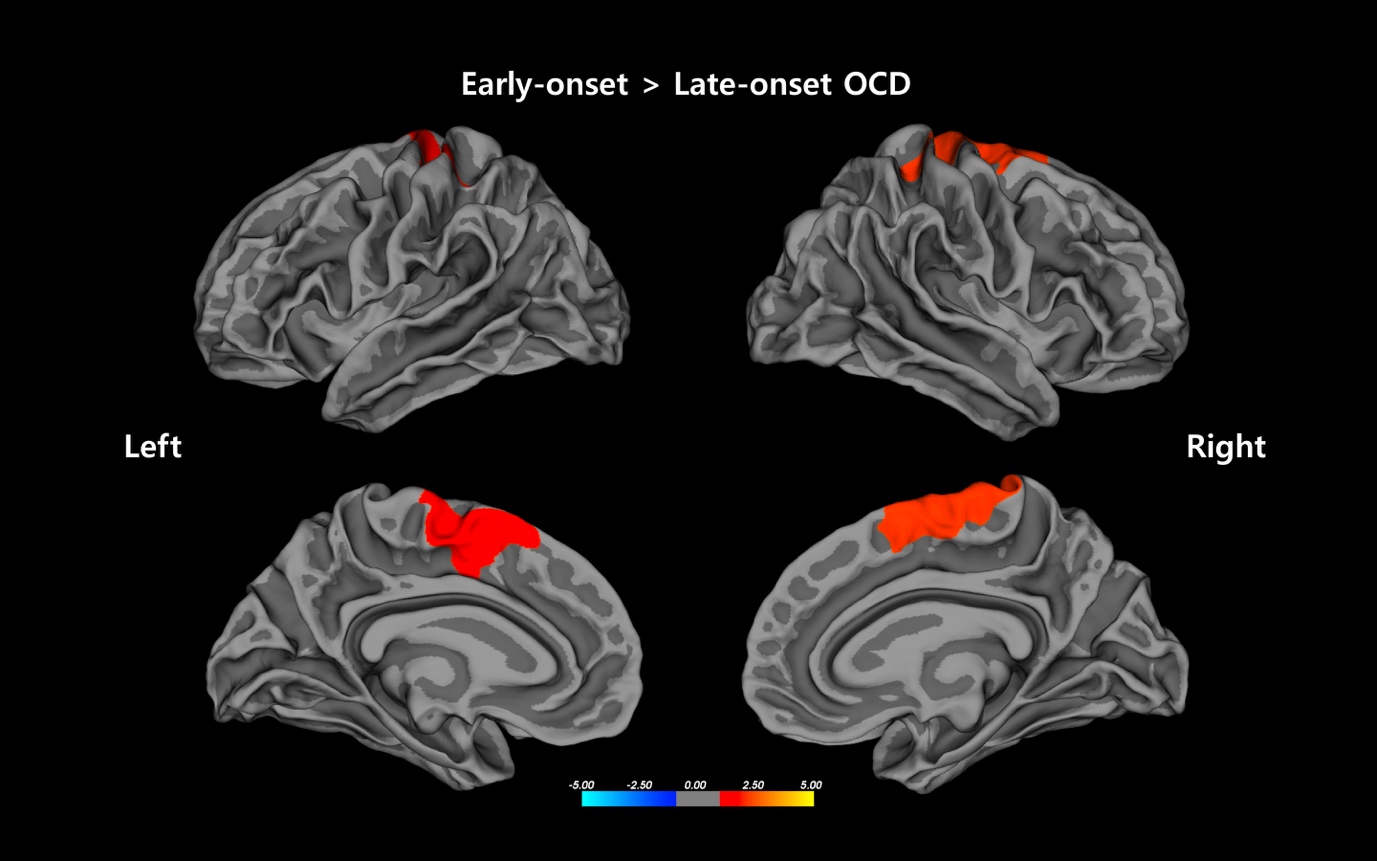


**Supplementary Tables**

**Table S1**. Description of clusters with a significantly increased local gyrification index (lGI) in patients with early-onset obsessive–compulsive disorder (OCD) compared to patients with late-onset OCD in the left and right hemispheres controlling for both age and duration of illness (DOI) as covariates smoothed with a full-width at half-maximum (FWHM) of 5 mm after clusterwise correction for multiple comparisons using Monte Carlo simulation (*p* < 0.05).

| Cluster  number | Peak vertex  cluster | VtxMax | Size  (mm2) | Peak vertex MNI  x y z | | | CWP (*p*) |
| --- | --- | --- | --- | --- | --- | --- | --- |
| 1 | L superior frontal | 133896 | 2160.85 | -6.6 | -2.7 | 51.8 | < 0.05* |
| 2 | R precentral | 14068 | 2636.07 | 11.0 | -14.8 | 66.1 | < 0.01** |

*Abbreviations:* L, left hemisphere; R, right hemisphere; VtxMax, number of peak vertices in the significant cluster; MNI, Montreal Neurological Institute (coordinate system); CWP, clusterwise probability and the nominal p value; * *p* < 0.05; ***p* < 0.01.
